# The Fight against Poliovirus Is Not Over

**DOI:** 10.3390/microorganisms11051323

**Published:** 2023-05-17

**Authors:** Chaldam Jespère Mbani, Magloire Pandoua Nekoua, Donatien Moukassa, Didier Hober

**Affiliations:** 1 Laboratoire de Virologie URL3610, Université de Lille, CHU Lille, 59000 Lille, France; mbanichaldam27@gmail.com (C.J.M.); magloire-pandoua.nekoua@univ-lille.fr (M.P.N.); 2 Laboratoire de Biologie Cellulaire et Moléculaire, Faculté des Sciences et Technique, Université Marien Ngouabi, Brazzaville BP 69, Congo; donatienmoukassa@gmail.com

**Keywords:** poliovirus, vaccine, reversion, vaccine-derived poliovirus, eradication

## Abstract

Poliovirus (PV), the virus that causes both acute poliomyelitis and post-polio syndrome, is classified within the *Enterovirus C* species, and there are three wild PV serotypes: WPV1, WPV2 and WPV3. The launch of the Global Polio Eradication Initiative (GPEI) in 1988 eradicated two of the three serotypes of WPV (WPV2 and WPV3). However, the endemic transmission of WPV1 persists in Afghanistan and Pakistan in 2022. There are cases of paralytic polio due to the loss of viral attenuation in the oral poliovirus vaccine (OPV), known as vaccine-derived poliovirus (VDPV). Between January 2021 and May 2023, a total of 2141 circulating VDPV (cVDPV) cases were reported in 36 countries worldwide. Because of this risk, inactivated poliovirus (IPV) is being used more widely, and attenuated PV2 has been removed from OPV formulations to obtain bivalent OPV (containing only types 1 and 3). In order to avoid the reversion of attenuated OPV strains, the new OPV, which is more stable due to genome-wide modifications, as well as sabin IPV and virus-like particle (VLP) vaccines, is being developed and offers promising solutions for eradicating WP1 and VDPV.

## 1. Introduction

Poliovirus (PV) is one of the best-characterized positive-strand RNA viruses that belongs to the *Enterovirus* genus of the *Picornaviridae* family. PV is classified within the *Enterovirus C* species, and there are three wild PV serotypes: WPV1, WPV2 and WPV3 [1]. Enteroviruses are mainly transmitted by the faecal–oral and respiratory routes. They initially replicate in the gastrointestinal or respiratory epithelium and can then spread to other tissues and organs via the lymphatic system and bloodstream [2]. PV can reach the central nervous system (CNS), mainly the spinal cord, destroying motor neurons and causing acute paralytic poliomyelitis [3,4]. Since the Global Polio Eradication Initiative (GPEI) was launched in 1988, two of the three serotypes of WPV—WPV2 and WPV3—were eradicated in 2015 and 2019, respectively [5]. However, the endemic transmission of WPV1 persists in Afghanistan and Pakistan in 2022 [5]. Nine cases of paralytic polio caused by WPV1 in children and adolescents were reported in Southeast Africa between November 2021 and December 2022, including one case in Malawi and eight cases in Mozambique [6]. The oral poliovirus vaccine (OPV) has protected millions of children from paralysis by preventing person-to-person transmission of the virus, which is essential for polio eradication [7]. Due to the variability and rapid evolution of enteroviruses, polio eradication may be more difficult than initially expected [8]. A long-standing disadvantage of using OPVs is that attenuated vaccine strains can revert to a neuropathogenic phenotype during their replication in the intestinal tract [9,10,11,12,13,14]. In communities with low vaccination coverage, these pathogenic OPV-derived strains (known as VDPV, vaccine-derived PV) can have the capacity to circulate in these popula-tions, maintain a reservoir of pathogenic strains and cause polio [15,16]. This review discusses the current epidemiological situation of WPV and VDPV and new strategies to fight against PV infections for the eradication of poliomyelitis.

## 2. Poliovirus

### 2.1. Structure and Pathogenesis

The PV consists of a positive-sense, single-stranded RNA genome of approximately 7.4 kb [17], like any other enterovirus. This genome is surrounded by an approximately 30 nm diameter icosahedral capsid consisting of 60 copies of each of four structural proteins (VP1, VP2, VP3 and VP4) [17]. The genome contains a large open reading frame (ORF), flanked by a highly structured 5′ untranslated region (5′ UTR) and a 3′ UTR terminated with a poly(A) tail [17]. The ORF encodes a polyprotein that is cleaved into four capsid proteins (VP1, VP2, VP3 and VP4) (structural proteins) and seven other proteins, 2A, 2B, 2C, 3A, 3B, 3C and 3D (non-structural proteins), involved in viral replication [17]. A second, shorter ORF (ORF2) translated into a single protein (ORF2p) was recently identified in human EVs [18,19].

**Figure 1:**
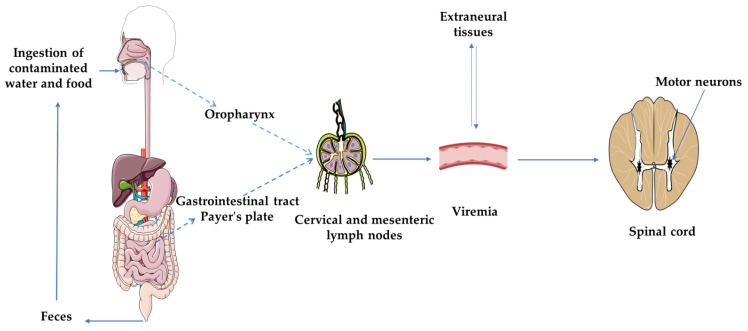
Pathogenesis of poliomyelitis. The poliovirus is transmitted via the fecal–oral route and reaches the intestinal tract after infecting the oropharynx and surviving the acidity of the stomach. It replicates in lymphoid tissues, such as the tonsils and Peyer’s patches, and is shed in stool for several weeks. From the primary replication sites, the virus spreads to regional lymph nodes (cervical and mesenteric) and the bloodstream, potentially infecting extraneural tissues and amplifying viremia. The poliovirus can breach the intestinal epithelium barrier and cross the blood–brain barrier to reach the central nervous system, where it targets motor neurons located in the ventral horns of the cervical and lumbar regions of the spinal cord.

PV has a limited host range, and humans are the only natural reservoir [20,21]. Transmission is mainly via the faecal–oral route, through contaminated food and water [22]. All three PV serotypes infect host cells by binding to a common cellular receptor called CD155 (or PV receptor, previously abbreviated PVR) [23]. Binding of PV to CD155 induces a conformational change in the viral capsid, which is essential for the release of viral RNA into the cytoplasm of infected cells. This process is known as decapsidation [24,25]. After infection, the virus first replicates in the pharynx and small intestine, particularly in the lymphoid tissues (tonsils and Peyer’s patches) [26]. PV then migrates to the regional lymph nodes (cervical and mesenteric) and into the bloodstream [27]. In some cases, the virus can reach the CNS by crossing the blood–brain barrier via infected mononuclear cells [28]. It has also been suggested that PV can reach the CNS through many peripheral nerve endings in muscles with passage along the nerve pathways to the CNS [29]. In the CNS, PV can destroy motor neurons in the anterior corona of the spinal cord, causing a characteristic flaccid paralysis without permanent sensory loss (Figure 1) [4]. The average incubation period for poliomyelitis is approximately 10 days (range 5–25 days) [30,31]. However, in some cases, the incubation period may be longer, up to years after vaccination with OPV [32]. It has been reported that multiple intramuscular injections administered during the incubation period of WPV infection or shortly after exposure to OPV increase the risk of paralytic poliomyelitis [33,34]. Less than 1% of polio cases result in temporary or permanent acute flaccid paralysis (AFP) [22]. Almost half of acute polio survivors develop post-polio syndrome (PPS) [35], which is the late onset or worsening of new disabling neuromuscular symptoms 30–40 years after initial polio [35]. PV, previously thought to be a lytic virus, can cause persistent infection in cell cultures of neural origin, as shown by several studies [36]. Furthermore, it can persist in the central nervous system of mice after the onset of paralysis, which is implicated in the pathogenesis of PPS [36,37]. Specific PV genomic sequences were detected in PPS patients, with the presence of mutations, particularly in the 5′ UTR and in the VP1 capsid region [36]. Other studies have suggested an inflammatory or autoimmune basis for PPS [37]. Indeed, inflammatory changes in the spinal cord have been observed postmortem in PPS patients [38,39]. In addition, levels of TNF-α and IFN-γ have been observed in the serum and cerebrospinal fluid of PPS patients [40,41].

### 2.2. Diagnosis

Cell culture is the primary method for the detection of poliovirus in clinical specimens [42,43]. Stool samples are the most suitable for the isolation of PV [44]. RD (a human rhabdomyosarcoma cell line) and HEp-2 cell lines were used for PV isolation and characterisation since they are easy to maintain. However, these lines can also support the growth of other non-polio enteroviruses [42]. Therefore, L20B (a mouse L cell line expressing the PV receptor) is currently preferred because it is more sensitive and selective for detecting PV [42,45,46]. Real-time RT-PCR (rRT-PCR) followed by sequencing is used to identify WPV serotype or VDPV and vaccine-like (Sabin-like) strains from cultures showing a viral cytopathic effect (CPE). This is known as intra-typical differentiation (ITD) [42,43,47,48]. Other methods include enzyme-linked immunosorbent assay (ELISA), which uses highly specific polyclonal cross-absorbing antisera to detect antigenic differences in OPV strains and RT-PCR followed by restriction fragment length polymorphism (RFLP) analysis [48,49,50]. It should be noted that ELISA and RFLP are no longer supported by WHO reagents and are no longer used [51].

The measurement of serum PV-neutralising antibody titres is a highly specific test for assessing protection against PV. The presence of antibodies at a dilution of at least 1:8 is considered a reliable indicator of protection [52,53]. In assessing the efficacy of polio vaccines, it is important to distinguish between humoral and mucosal immunity [53]. Humoral immunity is measured by the presence of PV-specific neutralising antibodies in the blood, mainly IgG, detected through a standardised microneutralisation test [54]. Mucosal immunity can be assessed by measuring the presence of PV-specific antibodies in nasopharyngeal samples, saliva or faeces. Tests include ELISA for PV-specific IgA and neutralisation tests [55]. Secretory immunity can be assessed through the measurement of circulating specific antibody secreting cells (ASCs) via ELISPOT [56,57,58]. ASCs produce antibodies of known antigenic specificity (e.g., vaccine antigen) [56]. The detection of ASCs in the blood as early as 5 days after vaccination or natural infection indicates recent exposure [56].

## 3. Paralytic Polio Vaccines and Eradication of WPV

**Figure 2:**
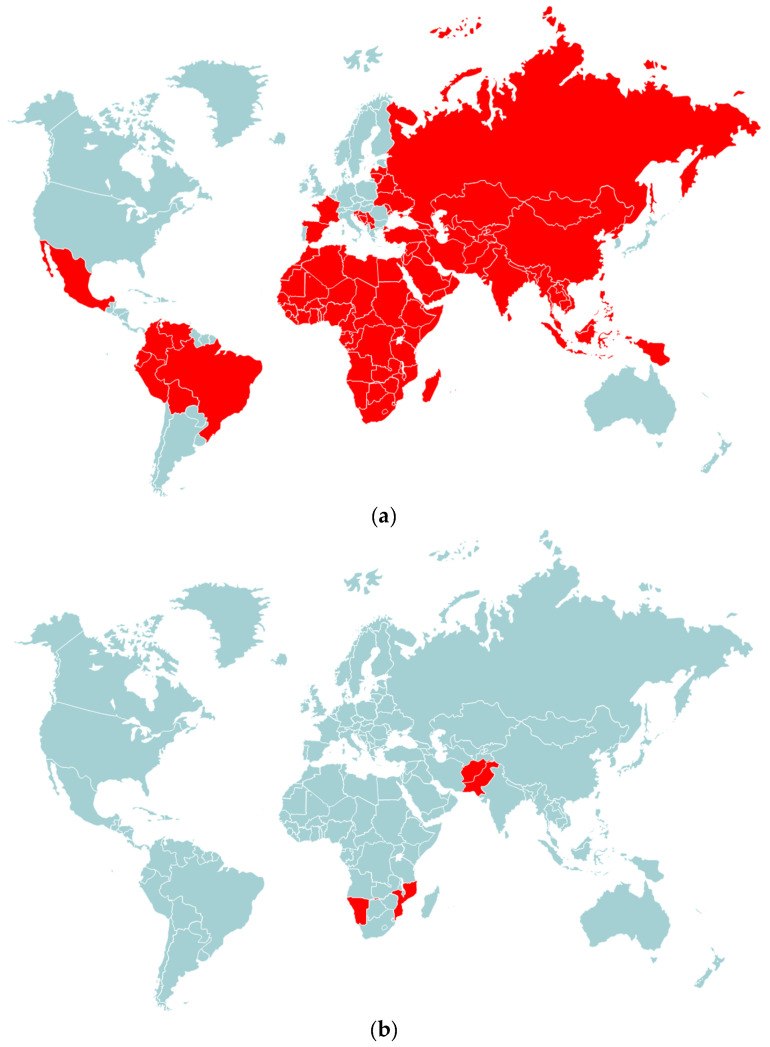
Wild poliovirus circulation in the world in 1988 and between January 2021 and May 2023. (**a**) In 1988, when the Global Polio Eradication Initiative (GPEI) was launched, polio paralyzed more than 350,000 children in 125 countries. (**b**) Between January 2021 and May 2023, only one of the three wild poliovirus serotypes, type 1 (WPV1), was circulating in only four countries: Afghanistan, Pakistan, Namibia and Mozambique.

The Global Polio Eradication Initiative (GPEI) has been working tirelessly towards the eradication of poliomyelitis since 1988. As a result of its efforts, progress has been impressive: the number of paralytic polio cases worldwide has been reduced by more than 99%, and the number of countries where the disease is endemic has fallen dramatically [59]. When the GPEI began, polio was endemic on all continents, affecting 125 countries with an estimated 350,000 cases per year and causing 5–10% mortality (Figure 2a). This means that approximately 1000 people were newly paralyzed, and between 50 and 100 people died of the disease every day worldwide [60,61]. To date, among the three serotypes of WPV, only WPV1 remains endemic in two countries in the world, Pakistan and Afghanistan [5]. The latest polio eradication report for the period January 2020 to June 2022 shows that although the COVID-19 pandemic has had an impact on polio immunisation campaigns, eradication efforts are ongoing [59]. In fact, compared with previous years, a sharp decline in WPV1 cases has been reported in Afghanistan and Pakistan [59]. In 2021, five cases of WPV1 were reported in these two countries, which represents a significant decrease compared to the previous year. One case of WPV1 was also reported in Namibia (non-endemic country) [6]. In 2022, 2 cases of WPV1 were reported in Afghanistan, 20 in Pakistan and 8 cases in Mozambique (non-endemic country) (Figure 2b) [6]. Only one case of WPV1 was reported in Pakistan between January 2023 and May 2023 [62].

Vaccines are the most effective way to prevent polio. The two types of vaccine used against the disease, injectable inactivated polio vaccine (IPV) and oral polio vaccine (OPV), have played a crucial role in reducing the number of cases [61,63]. OPV, also known as the Sabin vaccine, initially contained three live attenuated strains of PV. PV strains with reduced neurovirulence were developed using an attenuation technique of successive passages of the virus in non-human primates and in cultured primate cells [64]. OPV is a vaccine designed to mimic the natural immune response to WPV. It stimulates humoral and mucosal immunity [65]. There are three OPV formulations, each containing different combinations of Sabin PV strains for the three serotypes: tOPV (trivalent OPV), bOPV (bivalent OPV) and mOPV (monovalent OPV). tOPV, used for routine polio immunisation and mass campaigns in over 100 countries, has been gradually replaced by bOPV types 1 and 3 since 2016 due to the reversion of attenuated Sabin PV2 [66]. OPV vaccination is effective in preventing the shedding of WPV in the stool after exposure, which suggests that OPV induces sufficient intestinal mucosal immunity against PV infection and helps in the prevention of virus transmission [63]. It is considered cost-effective and effective in reducing WPV transmission in resource-constrained countries. OPV confers passive immunity to unvaccinated individuals through the attenuated vaccine strains shed in the feces of vaccinated individuals [65]. Efficacy may vary between populations. However, OPV vaccination has been shown to have high seroconversion rates for different poliovirus serotypes [66].

IPV is administered via intramuscular injection and contains a strain of each of the three serotypes inactivated with formalin and adsorbed into adjuvants [67]. IPV does not induce sufficient intestinal immunity to be effective in the prevention of virus spread [63,67]. IPV induces high and close-to-100% seroconversion rates for all three poliovirus serotypes after a three-dose series, although lower rates may be observed in the presence of high maternal antibodies [68]. Several studies have shown that IPV reduces the duration and rate of virus shedding in immunised individuals compared with unimmunised individuals, although IPV is not effective in preventing PV replication in the gut [69,70]. However, this reduction is much less than the reduction seen in people who have received OPV [71,72]. IPV alone has successfully eliminated polio transmission in some Northern European countries (Sweden, Finland, Iceland and the Netherlands). The high standards of hygiene and sanitation in these countries may explain this eradication [73]. Although IPV is considered safe, it is important to note that silent transmission of WPV can occur in countries using only IPV [74]. The administration of IPV can significantly increase mucosal immunity in children who have received OPV, and this effect may be greater than that of an additional dose of OPV. The efficacy of co-administration of IPV and OPV in a mixed schedule has been evaluated in clinical trials in India. The results showed that in children who had previously been vaccinated with OPV, the administration of IPV significantly reduced the proportion of subjects who shed virus after a first dose of OPV [75,76].

## 4. Emergence of Vaccine-Derived Poliovirus Strains

The OPV has been essential in eradicating wild-derived poliomyelitis worldwide by inducing high intestinal immunity to prevent human-to-human transmission of poliovirus in communities [77]. However, OPV strains can mutate and evolve into VDPV, which may be neurovirulent in vaccinated individuals due to their intrinsic genetic instability [78,79,80,81]. A number of studies have identified three specific codon positions that are involved in this reversion, two in the 5′ UTR region and one in the VP1 region [82,83]. This instability is caused by the lack of a corrective function in RNA-dependent RNA polymerase, leading to point mutations occurring [83,84]. VDPV can be divided into three distinct categories.

The first type is circulating VDPV (cVDPV), which is spread from person to person in a community with a low level of polio immunisation coverage. The second type is immunodeficiency-associated VDPV (iVDPV), which is isolated from people with primary immunodeficiency disease (PID). Finally, the third type is ambiguous VDPV (aVDPV), which is either a clinical isolate from persons with unknown immunodeficiency or a wastewater isolate whose ultimate source is unknown [85,86,87]. Among the different categories of VDPVs, cVDPVs are currently the greatest public health concern [87,88]. Outbreaks of cVDPVs can be caused by all three OPV serotypes. However, cVDPV2 is most associated with cVDPV outbreaks. It is a widespread cVDPV and is responsible for most cases of AFP [89]. It has been shown that cVDPV strains are recombinant. Indeed, their genome consists of a capsid protein-coding region from the vaccine strain, while the non-structural protein-coding region—and sometimes one or both non-coding regions—is from other enteroviruses of the same *Enterovirus C* species [83]. The emergence of these recombinant viruses implies that the same host has been infected with both PV and an *Enterovirus C*, allowing both viruses to replicate simultaneously in host cells. These cVDPVs have acquired the important biological properties of the WPV, namely efficient transmission from person to person and the ability to cause severe paralysis [90]. The first outbreak of cVDPV was identified in the Dominican Republic and Haiti between 2000 and 2001. Affected patients were either partially vaccinated or unvaccinated [91]. New outbreaks of cVDPV have been reported later in several countries, including the Philippines, China, Indonesia, Cambodia, Madagascar, Myanmar and Niger, with the emergence of recombinant strains of cVDPV [92,93,94,95,96]. A severe outbreak of cVDPV2 occurred in Nigeria from 2005 for more than six years, with more than 300 cases of polio [97,98,99]. In general, the VP1 sequences examined from cVDPV isolates differed from parental OPV strains by only 1–3.5%, suggesting that reversion to the attenuated phenotype of Sabin strains occurs with circulation times of 1–4 years [91,92,93,95,100]. Strikingly, the cVDPVs involved in most of the above epidemics were recombinant strains, the product of genetic exchange between OPV strains and other *Enterovirus C* [97,101,102]. For example, recombinant cVDPV2 was involved in the polio outbreaks in India and the Democratic Republic of Congo between 2008 and 2010 [103]. Between January 2021 and May 2023, a total of 2141 cVDPV cases were reported in 36 countries worldwide [104]. The cases (both AFP and non-AFP) are divided into 269 cVDPV1 cases in 6 different countries, 1868 cVDPV2 cases in 35 different countries and 4 cVDPV3 cases in Israel. The number of AFP cases was 1629, of which 225 were associated with cVDPV1, 1403 with cVDPV2 and 1 with cVDPV3 [104]. The number of cases that was not associated with AFP was 512. cVDPVs have also been detected in 1055 environmental samples (Table 1) [104]. Immunocompetent individuals clear attenuated vaccine virus from their bodies after a limited period of shedding, unlike immunocompromised individuals, who may be unable to clear the intestinal replication of the vaccine virus, resulting in persistent intestinal poliovirus infection and prolonged shedding [105,106]. The persistence of these viruses in immunocompromised individuals may compromise polio eradication efforts. These individuals may represent a potential source of virus reintroduction once the disease is eradicated [107,108].

Between 1962 and 2016, there were 110 cases of iVDPV with onset of excretion or paralytic symptoms, including 101 cases with prolonged or chronic iVDPV excretion [106]. Since 2000, the number of reported cases has increased, particularly in low- and middle-income countries, while the number of reported cases has decreased in high-income countries [106]. From July 2018 to December 2019, 16 new iVDPV cases were reported in Argentina, Egypt, Iran, Philippines and Tunisia, including 8 iVDPV1, 7 iVDPV3 and 1 iVDPV2 [86] (Table 2). In March 2022, one case of iVDPV3 was detected in a stool sample from an infant with PID in China [109] (Table 2).

Between January 2017 and June 2018, there were 28 aVDPV cases in seven countries; 23 were aVDPV2, 4 were aVDPV3 and 1 was aVDPV1. These were primarily isolated from environmental specimens [110] (Table 2).

## 5. New Strategies to Fight against PV

For many years, the eradication of WPV has been a global public health goal. Complete eradication of poliomyelitis remains a major challenge, although significant progress has been made in controlling the disease. Immunisation is one of the most effective ways to prevent polio. The GPEI has two main goals: first, to end polio virus transmission in Afghanistan and Pakistan, the last two countries where wild poliovirus is endemic; second, to prevent outbreaks of cVDPV transmission in non-endemic countries. To achieve these goals, the GPEI will take a region- and country-specific approach [111]. New, safe and effective PV vaccines are urgently needed to achieve the complete eradication of the virus and to prevent possible re-emergence of the virus in a post-polio world.

Currently, new formulations of oral and inactivated vaccines are being developed. A new IPV has been licensed in Denmark and will soon be available on the global market. This vaccine contains an alum adjuvant and has only one-tenth of the antigen content of the conventional IPV [112]. Interestingly, a double-mutant labile toxin (dmLT) derived from *Escherichia coli* is a potent adjuvant that can enhance immune responses to IPV [113,114,115,116]. DmLT has been extensively studied for safety and immune efficacy in humans and animals. It has been administered alone or in combination with other vaccines through oral, sublingual, intramuscular and intradermal routes [114,115,117,118,119]. The World Health Organisation (WHO) approved the use of a fractional dose of IPV (fIPV), equivalent to one-fifth of the full dose given via intramuscular injection for routine immunisation, epidemic response and supplementary immunisation activities in response to shortages of trivalent IPV that can compromise poliovirus control. This method has been shown to be safe and effective, inducing immune responses comparable to those seen with a full dose of IPV. Several countries, including India, Nepal, Cuba, Bhutan and Ecuador, have begun to include fIPV in their immunisation programmes [120]. fIPV adjuvated with dmLT was well tolerated and induced systemic immune responses against all three WPV serotypes in a randomised phase 1 trial. This suggests that this approach may be useful in improving the effectiveness of polio vaccination programs around the world [121].

Improving the stability and safety of OPV vaccine strains is another goal of current polio vaccine research. To stabilise attenuation determinants, prevent recombination and limit viral adaptability, one approach is to introduce specific modifications into the genome of the Sabin PV2 strain [11,122]. Modifications have been made to certain regions of the virus genome to improve stability and prevent recombination and adaptability. Specifically, modifications are introduced into the 5′UTR region of the genome to stabilize attenuation determinants into the 2C coding region to prevent recombination and into the 3D polymerase coding region to limit viral adaptability [11]. A new attenuated polio vaccine (nOPV2) has been developed using this approach. Clinical trials have recently been conducted to assess the safety and efficacy of two nOPV2 vaccine candidates (nOPV2-c1 and nOPV2-c2). OPV2-c1 has been engineered to provide improved fidelity and reduced recombination by genetically stabilising its V region (the main Sabin 2 attenuating site), relocating its cis-acting replication element and modifying its polymerase. Similarly, nOPV2-c2 has the same genetically stabilised V region and a capsid coding region optimised at the codon level [11]. The safety and efficacy of nOPV2 (nOPV2-c1 and nOPV2-c2) have recently been evaluated in clinical trials. Designed to prevent cases of VDPV, both new vaccines have been developed. Phase 2 trials showed that the new vaccines were well tolerated and induced an immune response like that of the existing monovalent vaccine in an adult population with a history of OPV vaccination, as well as in children and infants [123,124]. nOPV2 is effective in inducing an immune response in children and can be used to eliminate WPV2 transmission, according to a study conducted in Tajikistan in 2021 [125]. nOPV2 was well tolerated and did not cause any serious adverse events in infants, according to results from a randomised phase 2 trial. Antibody levels were significantly higher in infants who received the vaccine compared to the placebo group. However, the duration of fecal shedding of the vaccine virus was longer in vaccinated infants than in the placebo group [126]. Burundi and the Democratic Republic of Congo (DRC) have reported the first cases of cVDPV2 associated with nOPV2. The viruses were isolated from stool samples of seven children with AFP (six in DRC and one in Burundi) and from five environmental samples collected in Burundi. It should be noted that, to date, nearly 600 million doses of nVPO2 have been administered in 28 countries around the world, and the majority of countries have experienced no further cVDPV2 transmission after two rounds of vaccination [127]. In a study, the efficacy of the inactivated Sabin strain vaccine (sIPV) was evaluated in response to an outbreak of cVDPV2 in the province of Sichuan, China, from 2019 to 2021. The results showed that the mass vaccination campaign with sIPV increased vaccination coverage and induced a strong immune response against cVDPV2. In addition, the circulation of cVDPV2 was rapidly suppressed [128].

Virus-like particles (VLPs) have been proposed as an alternative to traditional vaccines based on the inactivation of infectious virions [129]. A VLP is a particle that morphologically resembles a virus, preferably retaining its original antigenic structure. However, unlike a virus, it does not contain genetic material and cannot replicate [130]. In recent years, PV VLPs with native antigenicity have been produced in various systems, including mammalian cells, plants, insects, bacteria and yeast [129,131,132,133,134,135,136]. The viral particles produced were characterised in terms of morphology, size, stability and immunogenicity. The results showed that the viral particles produced were similar in morphology and size to natural viruses and were also stable at high temperatures. Immunological tests showed that the virus particles induced a strong immune response in vaccinated mice [136]. Co-expression of the poliovirus P1 capsid protein precursor and the 3CD protease in different systems has been shown to be efficient in the production of VLP that can induce a protective antibody response [137,138]. A recent study reported that remdesivir treatment eliminated iVDPV in the stool of a 50-year-old patient. These results suggest that remdesivir may be effective in the treatment of iVDPV [139].

## 6. Conclusions

Significant progress in reducing poliovirus incidence and transmission has been made since 1988. However, the goal of complete eradication remains elusive due to a number of factors, including the persistence of transmission foci in some parts of the world and difficulties in implementing mass immunisation programmes. Although two of the three serotypes of WPV have been certified as eradicated, WPV1 transmission remains active in some countries, particularly Afghanistan and Pakistan. In addition, outbreaks of VDPV, facilitated by the use of OPV, have occurred in several countries. This highlights the need to develop new strategies, such as innovative surveillance and the development of new vaccines, to support eradication goals. In conclusion, while progress has been made in the fight against polio, there must be no let-up in the pursuit of complete eradication. This will require continued international cooperation and effective coordination of immunization programmes in areas where the disease is still endemic.

## Figures and Tables

**Table 1:** Number of circulating VDPV (cVDPV) cases worldwide between January 2021 and May 2023.

VDPV	Countries	Number of Cases		Environmental Samples
		AFP Cases	Non-AFP Cases	
**cVDPV1**				
	Madagascar	36	37	168
	Malawi	4	1	0
	DRC	156	5	0
	Mozambique	25	1	0
	Congo	1	0	
	Yemen	3	0	
**cVDPV2**				
	Benin	16	3	12
	Burkina Faso	2	0	1
	Guinea	6	0	2
	Guinea-Bissau	3	1	0
	Burundi	1	2	13
	Botswana	0	0	5
	Algeria	3	5	26
	Central African Republic	10	1	9
	Cameroon	6	3	1
	Chad	50	7	6
	Côte d’Ivoire	1	0	3
	Democratic Republic of the Congo	412	37	13
	Congo	2	0	3
	Eritrea	2	0	0
	Ethiopia	11	0	0
	Ghana	3	4	19
	Mauritania	0	4	7
	Mozambique	6	0	0
	Malawi	0	0	1
	Niger	33	4	15
	Nigeria	467	234	397
	Senegal	17	34	15
	Zambia	0	0	3
	United States of America	1	0	14
	Canada	0	0	2
	Djibouti	0	0	19
	Egypt	0	0	18
	Mali	2	0	0
	Somalia	8	4	7
	Soudan	1	0	1
	Yemen	228	50	38
	Israel	1	0	55
	United kingdom	0	0	6
	Ukraine	2	18	0
	Indonesia	4	10	0
	Uganda	0	0	2
	Gambia	0	0	9
	Pakistan	8	0	35
	Tajikistan	35	22	17
	Afghanistan	43	2	40
	Sierra Leone	5	8	9
	Liberia	3	5	14
	South Sudan	9	5	0
	Iran	0	0	0
	Kenya	0	2	1
	Togo	2	0	2
**cVDPV3**				
	Israel	1	3	30
	Occupied Palestinian territory	0	0	16
	China	0	0	1
TOTAL		1629	512	1055

VDPV: vaccine-derived poliovirus; cVDPV: circulating VDPV; AFP: acute flaccid paralysis.

**Table 2:** Number of iVDPV and aVDPV cases worldwide from July 2018 to December 2019 and from January 2017 to June 2018, respectively.

VDPV	Countries	Years	Number of Cases		Environmental Samples
iVDPV			AFP	Non-AFP	
iVDPV1	Egypt	2018–2019	1	4	0
	Iran	2018–2019	2	1	0
iVDPV2	Philippines	2018–2019	1	0	0
iVDPV3	Egypt	2018–2019	1	4	0
	Tunisia	2018–2019	1	0	0
	Argentina	2018	1	0	0
TOTAL			7	9	0
**aVDPV**					
aVDPV1	Democratic Republic of the Congo	2017–2018	1	0	0
aVDPV2	Nigeria	2017–2018	0	1	12
	Pakistan	2017–2018	0	0	5
	Somalia	2017–2018	0	0	1
	India	2017–2018	0	0	1
	Democratic Republic of the Congo	2017–2018	2	0	0
	Australia	2017–2018	0	0	1
aVDPV3	China	2017–2018	1	0	2
	India	2017–2018	0	0	1
TOTAL			4	1	23

VDPV: vaccine-derived poliovirus; iVDPV: immunodeficiency-associated VDPVs; aVDPV: ambiguous VDPV.
